# Efficacy and safety of [^161^Tb]Tb-PSMA radioligand therapy in metastatic castration resistant prostate cancer: a systematic review and meta-analysis

**DOI:** 10.3389/fmed.2026.1925657

**Published:** 2026-08-05

**Authors:** Domenico Albano, Gaetano Paone, Giorgio Treglia

**Affiliations:** 1 Nuclear Medicine, University of Brescia, Brescia, Italy; 2 Nuclear Medicine Department, ASST Spedali Civili di Brescia, Brescia, Italy; 3 Division of Nuclear Medicine, Ente Ospedaliero Cantonale, Bellinzona and Lugano, Switzerland; 4 Faculty of Biomedical Sciences, Università della Svizzera italiana, Lugano, Switzerland; 5 Faculty of Biology and Medicine, University of Lausanne, Lausanne, Switzerland

**Keywords:** meta-analysis, nuclear medicine, prostate cancer, PSMA, radioligand therapy, Tb-161

## Abstract

**Introduction:**

Prostate-specific membrane antigen (PSMA)-targeted radioligand therapy (RLT) using Terbium-161 [^161^Tb]-labeled compounds has recently been investigated in patients with metastatic castration-resistant prostate cancer (mCRPC). We performed a systematic review and meta-analysis to evaluate the efficacy and safety of [^161^Tb]Tb-PSMA RLT in this clinical setting.

**Methods:**

We conducted a comprehensive literature search across PubMed/MEDLINE, Embase, and the Cochrane databases to retrieve published articles on the efficacy and safety of [^161^Tb]Tb-PSMA RLT in mCRPC. Studies reporting the use of [^161^Tb]Tb-PSMA RLT in mCRPC patients were eligible for inclusion. Pooled estimates of disease control rate (DCR), objective response rate (ORR), and adverse event rate were calculated using a random-effects model. Statistical heterogeneity was assessed using the I^2^ statistic.

**Results:**

Six studies published between 2024 and 2026, including 80 mCRPC patients treated with 291 cycles of [^161^Tb]Tb-PSMA RLT, were included. Most patients had pretreated disease and had previously received PSMA-targeted radionuclide therapy. The pooled DCR was 83.9% (95% confidence interval [CI]: 76.0–92.0%) with no significant heterogeneity (I^2^ = 0%). The pooled ORR was 50.2% (95% CI: 27.0–74.0%), although significant heterogeneity was observed (I^2^ = 81%). Across all studies, 124 adverse events were reported, corresponding to a pooled adverse event rate of 41%, but high grade were only 18.

**Conclusion:**

Current evidence suggests that [^161^Tb]Tb-PSMA RLT is a promising therapeutic option for patients with mCRPC, demonstrating encouraging disease control and an acceptable safety profile. Nevertheless, defining its precise role requires larger-scale, comparative trials conducted across multiple centers over longer observation periods.

## Introduction

Prostate cancer (PCa) is the second most common type of cancer diagnosed in men and the fifth leading cause of death (1). While global prostate cancer incidence continues to climb, mortality rates in many high-income nations have stabilized or decreased, a trend largely credited to improved screening protocols and more effective systemic therapies. Nevertheless, real-world data show that approximately 20 to 30% of patients initially diagnosed with hormone-sensitive disease will eventually progress to metastatic castration-resistant prostate cancer (mCRPC), typically within a few years.

Over the recent decades, the therapeutic landscape for metastatic castration-resistant prostate cancer (mCRPC) has expanded to incorporate radioligand therapy (RLT) targeting prostate-specific membrane antigen (PSMA). Notably, Lutetium-177 [^177^Lu]-PSMA-617 RLT has been shown to significantly prolong progression-free survival while maintaining a favorable safety profile, particularly in patients with large-volume lesions (2, 3). However, the beta-particles emitted by [Lu177] possess modest energy and a limited tissue penetration range, which may render them less effective at eradicating microscopic disease. This limitation underscores the need for novel radionuclides capable of targeting microlesions more efficiently. Terbium-161 ([^161^Tb]) has emerged as a promising therapeutic radionuclide; while it emits beta-particles similar to [^177^Lu] it offers a distinct therapeutic advantage by additionally emitting low-energy Auger and internal conversion electrons, which facilitate the targeted destruction of micro-metastases (4–6). Consequently, there is growing interest in utilizing [^161^Tb] either as an alternative or a complementary agent to [^177^Lu].

To address this, the objective of this systematic review and meta-analysis is to evaluate the clinical efficacy and safety of [^161^Tb]Tb-PSMA RLT in patients with mCRPC.

## Methods

### Protocol

This systematic review and meta-analysis was conducted in accordance with a predetermined protocol and guided by the Preferred Reporting Items for Systematic Reviews and Meta-Analyses (PRISMA 2020) statement (7). The completed PRISMA checklist is provided in Supplementary Table 1. Pre-registering was not carried out according to PRISMA statement (item 24). As a first step, a direct review query using the Population, Intervention, Comparator, and Outcomes (PICO) framework was executed: “What is the efficacy and safety (“outcome”) of [^161^Tb]Tb-PSMA RLT (“intervention”) in patients with mCRPC (“population”) in comparions or not with other treatments (“comparator”)?.” Two investigators (D. A. and G. T.) independently conducted the literature search, study selection, data extraction, and quality assessment. Any discrepancies or disagreements were resolved through consensus and mutual discussion.

### Search strategy

We did a research on the PubMed/MEDLINE, Embase and Cochrane databases to find pertinent published articles about the role of [^161^Tb]-PSMA RLT in patients affected by mCRPC. We searched for relevant literature up to June 15, 2026, with no start-date restrictions. The search strategy combined the terms (1) “Terbium” OR “Tb” and (2) “prostate” OR “prostatic.” To expand our search, we manually screened the reference lists of all retrieved articles to identify any further relevant publications.

### Study selection process

Studies were excluded if they met any of the following criteria: (1) records outside the scope of interest; (2) review articles, meta-analyses, letters, conference proceedings, and editorials; (3) case reports or small case series involving fewer than four patients, to minimize selection bias from underpowered studies; or (4) non-human studies.

Two investigators (G. T. and D. A.) independently screened the titles and abstracts of the retrieved records against these predefined inclusion and exclusion criteria. Subsequently, the same two reviewers independently evaluated the full-text articles of potentially eligible studies to determine their final suitability for inclusion. Considering the rarity of this condition, we included articles with at least 4 patients treated with [^161^Tb]-PSMA RLT.

### Data extraction process and collection

For each included study, data were extracted regarding baseline study characteristics (first author, year of publication, country of origin, funding source, and study design) and clinical variables (including sample size, patient age, baseline prostate-specific antigen [PSA] levels, and clinical setting). Additionally, we compiled technical parameters concerning the radiopharmaceutical characteristics and treatment protocols, alongside primary therapeutic outcomes such as biochemical response and safety profiles. These findings are summarized in the accompanying tables and described in detail within the Results section.

### Quality assessment (risk of bias assessment)

The methodological quality of the included studies was independently evaluated by two investigators (G. T. and D. A.) utilizing the 14-item National Institutes of Health (NIH) Quality Assessment Tool for Observational Cohort and Cross-Sectional Studies (8). Any discrepancies in assessment were resolved through consensus. Based on their final scores, the studies were categorized into three quality tiers: good (9–14 points), fair (5–8 points), or poor (0–4 points).

### Statistics

For the efficacy of RLT we investigated the disease control rate (DCR) and the objective response rate (ORR). DCR was defined as (complete response + partial response + stable disease)/total number of patients. ORR was defined as (partial response + complete response)/total number of patients.

All pooled DCR and ORR values refer to biochemical (PSA-based) response according to the criteria used in each original study. Progressive disease (PD) was defined as a PSA value increasing ≥ 25% from baseline to follow-up. Partial response (PR) of disease was defined as the baseline PSA value decreasing ≥ 50%, and stable disease (SD) was defined as a PSA decrease of < 50% or an increase < 25%.

Pooled estimates and their corresponding 95% confidence intervals (CIs) were calculated using a random-effects model, as described by DerSimonian and Laird (9). Forest plots were generated to visualize the meta-analysis results. To evaluate statistical heterogeneity, we utilized the $I^2$ index (inconsistency index) (9). Publication bias was assessed qualitatively via visual inspection of funnel plots and quantitatively using Egger’s test. All statistical analyses were conducted using the open-source OpenMetaAnalyst software, developed by the Center for Evidence Synthesis in Health at Brown University (USA).

## Results

### Literature search and study selection

**Figure 1:**
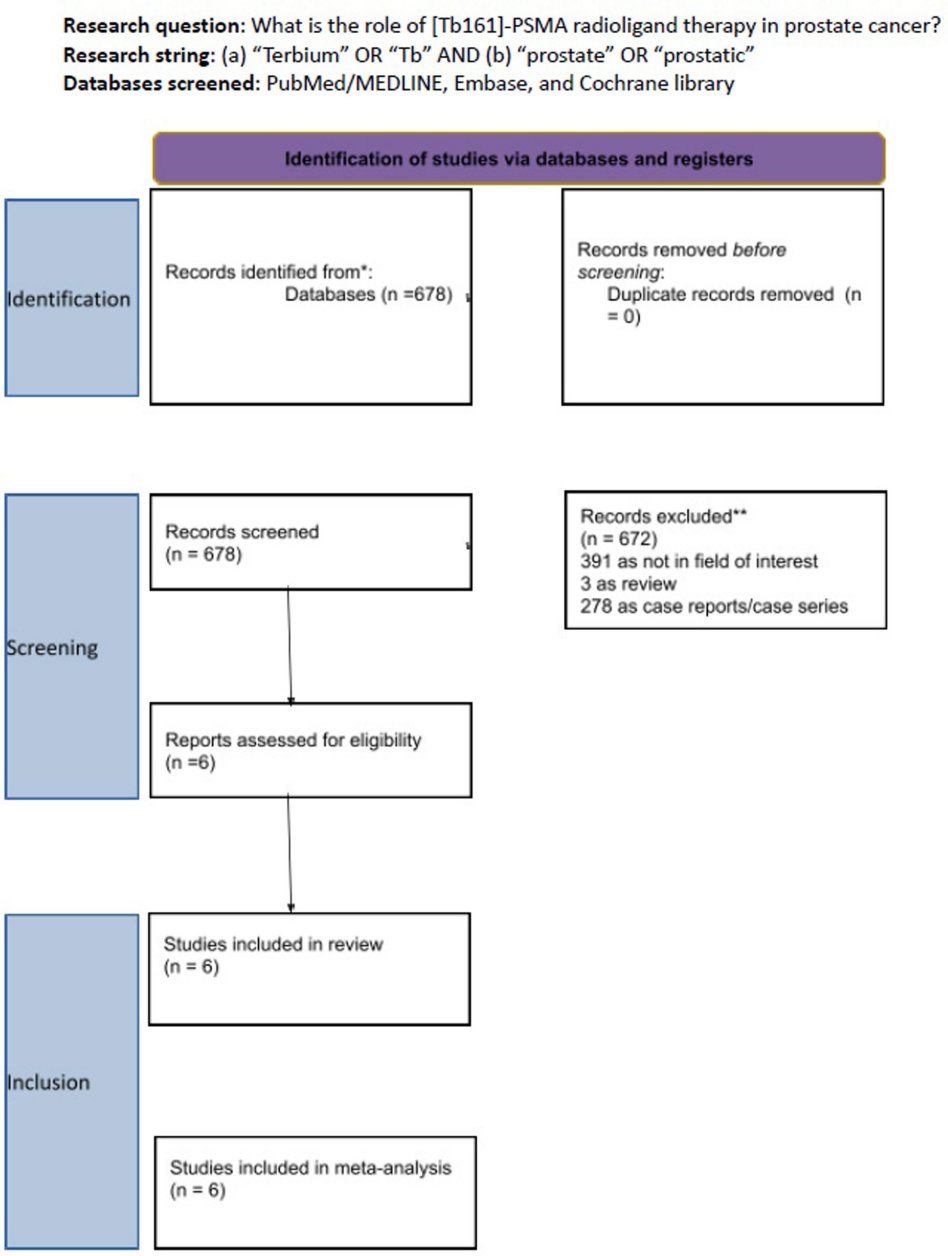
Literature search flowchart.

Our literature search, last updated on June 15, 2026, initially yielded 678 records. After screening titles and abstracts against our selection criteria, 672 records were excluded. The primary reasons for exclusion were: 391 records fell outside the field of interest; three were reviews, letters, or editorials; and 278 were case reports or small case series. Ultimately, six articles (10–15) met the eligibility criteria and underwent full-text assessment, with all six subsequently included in both the qualitative synthesis (systematic review) and quantitative synthesis (meta-analysis). A manual search of the reference lists of these selected studies did not yield any additional relevant publications. The study selection process is illustrated in Figure 1.

### Studies and patients characteristics

Six studies between 2024 and 2026 were included in our analysis for a total of 80 patients. Four studies were prospective (12–15), while the remaining two were retrospective (10, 11). Half of the research was conducted in Germany. Median age of the patients included in these studies ranged from 64 to 78.1 years. Median PSA serum values before [^161^Tb]Tb-PSMA RLT among the included mCRPC patients ranged from 22.6 to 280 ng/mL (Table 1).

**Table 1:** Main features of studied included.

First author	Year	Country	Funding source	Study design	N° patients	Age median (range)	PSA median (range) ng/mL	Setting
Schaefer-Schuler et al. (10)	2024	Germany	None declared	R	6	74 (69–85)	280 (44–2,148)	mCRPC
Al-Ibraheem et al. (11)	2024	Jordan	None declared	R	4	64*	49.4 (9.5–117)	mCRPC
Buteau et al. (12)	2025	Australia	Yes	P	30	69 (66–74.8)	26.9 (10.1–70)	mCRPC
Rosar et al. (13)	2025	Germany	None declared	P	18	76 (65–87)	90 (0.4–474)	mCRPC
Kucuk et al. (14)	2025	Turkey	None declared	P	7	71 (53–82)	22.6 (3.6–7,873)	mCRPC
Rosar et al. (15)	2026	Germany	None declared	P	15	78.1 (67–88)	71 (0.4–312)	mCRPC

P, prospective; R, retrospective; mCRPC, metastatic castration-resistant prostate cancer.

*Mean.

A total of two radiotracers were included: [^161^Tb]Tb-PSMA-617 in four articles (10, 11, 13, 15) and [^161^Tb]Tb-PSMA-I&T in two articles (12, 14).

The protocol was very different between the studies. In two researches (10, 11) only one cycle of [^161^Tb]Tb-PSMA RLT was injected with a fixed activity 5.5 Gbq. In the other 4 studies (12–15), the number of cycles of RLT ranged from one to seven. In all studies except one, [^161^Tb]Tb-PSMA RLT was performed after [^177^Lu]-PSMa RLT or [225Ac]-PSMA RLT. Only Buteau et al. (12) treated patients with [^161^Tb]Tb-PSMA RLT after ARPI and taxane chemotherapy failure (Table 2).

**Table 2:** Main technical features of studied included.

First author	Radiopharmaceutical	Administration protocol	Biochemical response	Previous treatment
Schaefer-Schuler et al. (10)	^161^Tb-PSMA-617	One cycle (5.5 GBq)	PR 1 patient (17%) SD 3 patients (50%) PD 2 patients (33%)	After ^177^Lu-PRLT (*n* = 2) or ^177^Lu-PRLT + ^225^Ac-PRLT failure (*n* = 4)
Al-Ibraheem et al. (11)	^161^Tb-PSMA-617	One cycle (5.5 GBq)	PR 1 patient (25%) SD 2 patients (50%) PD 1 patient (25%)	After ^177^Lu-PRLT
Buteau et al. (12)	^161^Tb-PSMA-I&T	One to four cycles (3.1–9.1 GBq)	PR 25 patients (83%) PD 5 patients (17%)	After ARPI and taxane chemotherapy failure
Rosar et al. (13)	^161^Tb-PSMA-617	One to six cycles (4.4–7.4 GBq)	PR 7 patients (39%) SD 6 patients (33%) PD 5 patients (28%)	After ^177^Lu-PRLT or ^225^Ac-PRLT
Kucuk et al. (14)	^161^Tb-PSMA-I&T	Two cycles (7.4 GBq)	PR 4 patients (57%) SD 1 patient (14%) PD 2 patients (29%)	After ^177^Lu-PRLT
Rosar et al. (15)	^161^Tb-PSMA-617	Two to seven cycles (4.4–7.4 GBq)	PR 10 patients (66%) SD 4 patient (27%) PD 1 patients (7%)	After ^177^Lu-PRLT

PRLT, PSMA-target radioling therapy; ARPI, androgen receptor pathway inhibitor; PR, partial response; PD, progression of disease; SD, stable disease.

### Risk of bias and applicability

Methodological quality was assessed using the NIH Quality Assessment Tool. All six studies included in this systematic review and meta-analysis were rated as “good.” The detailed scoring breakdown for each study is provided in Supplementary Table 2.

### Safety profile

Across six studies involving 80 patients and a total of 291 [^161^Tb]Tb-PSMA RLT cycles, 124 adverse events were registered with a pooled adverse event rate of 43% (124/291 cycles). Anemia was the most frequent biochemical adverse event with 29 cases, low grade (G1-2) in 21 cases and high grade in 8 (G3-4). Fatigue was the most frequent clinical side effect with 16 cases reported in the literature (Table 3).

**Table 3:** Summary of adverse events reported in the literature.

First author	Anemia	Thrombocitopenia	Leukopenia	Xerostomia	Fatigue	Gastrointestinal toxicities	Bone pain flare	Nephrotoxicity
Schaefer-Schuler et al. (10)	G3 → 1	G2 → 1	G3 → 1	G1 → 1 G2 → 1	0	0	1	0
Al-Ibraheem et al. (11)	0	0	0	0	G1 → 1 G2 → 1	0	0	G1 → 1
Buteau et al. (12)	G1 → 16 G2 → 4	G1 → 6	G1 → 7 G2 → 4	G1 → 24	G1 → 12 G2 → 1	G1 → 14	G1 → 3 G3 → 1	G1 → 1
Rosar et al. (13)	G3 → 2 G4 → 2	G3 → 2	0	0	0	0	0	G3 → 4
Kucuk et al. (14)	G1 → 1	0	0	0	G1 → 1	0	G2 → 1	0
Rosar et al. (15)	G3 → 2 G4 → 1	0	0	0	0	0	0	G3 → 6

G, grading.

### Meta-analysis (quantitative analysis)

**Figure 2:**
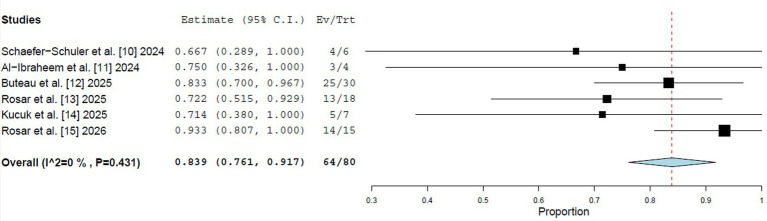
Forest plot of disease control rate for the assessment of biochemical efficacy of [^161^Tb]Tb-PSMA RLT.

**Figure 3:**
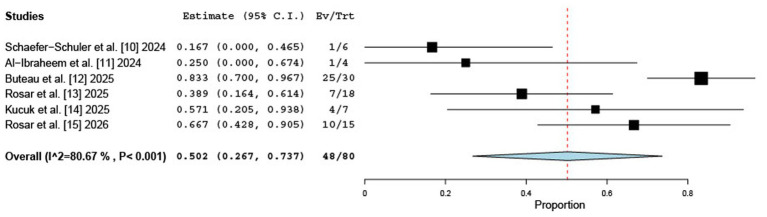
Forest plot of objective response rate for the assessment of biochemical efficacy of [^161^Tb]Tb-PSMA RLT.

**Figure 4:**
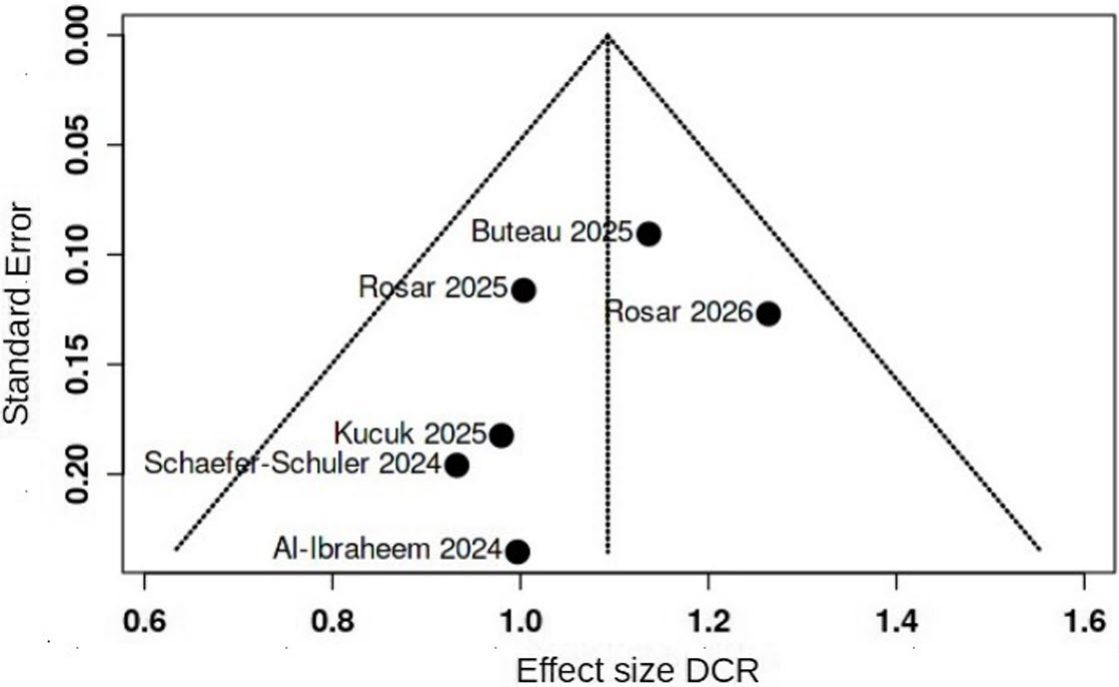
Funnel plot for the evaluation for bias assessment of studies concerning disease control rate for the assessment of biochemical efficacy of [^161^Tb]Tb-PSMA RLT.

Six studies reported outcomes following 291 [^161^Tb]Tb-PSMA cycles administered to 80 patients. The pooled DCR was 83.9% (95% CI: 76–92%) without significant heterogeneity (I^2^ = 0%, *p* = 0.431) (Figure 2). The pooled ORR was 50.2% (95% CI: 27–74%) with a significant heterogeneity (I^2^ = 81%, *p* < 0.001) (Figure 3). The funnel plot for DCR does not indicate a potential publication bias (Figure 4). The Egger’s test does not support the presence of funnel plot asymmetry (intercept: −1.31, 95% CI: −3.44–0.81, *t*: −1.211, *p*-value: 0.293).

**Figure 5:**
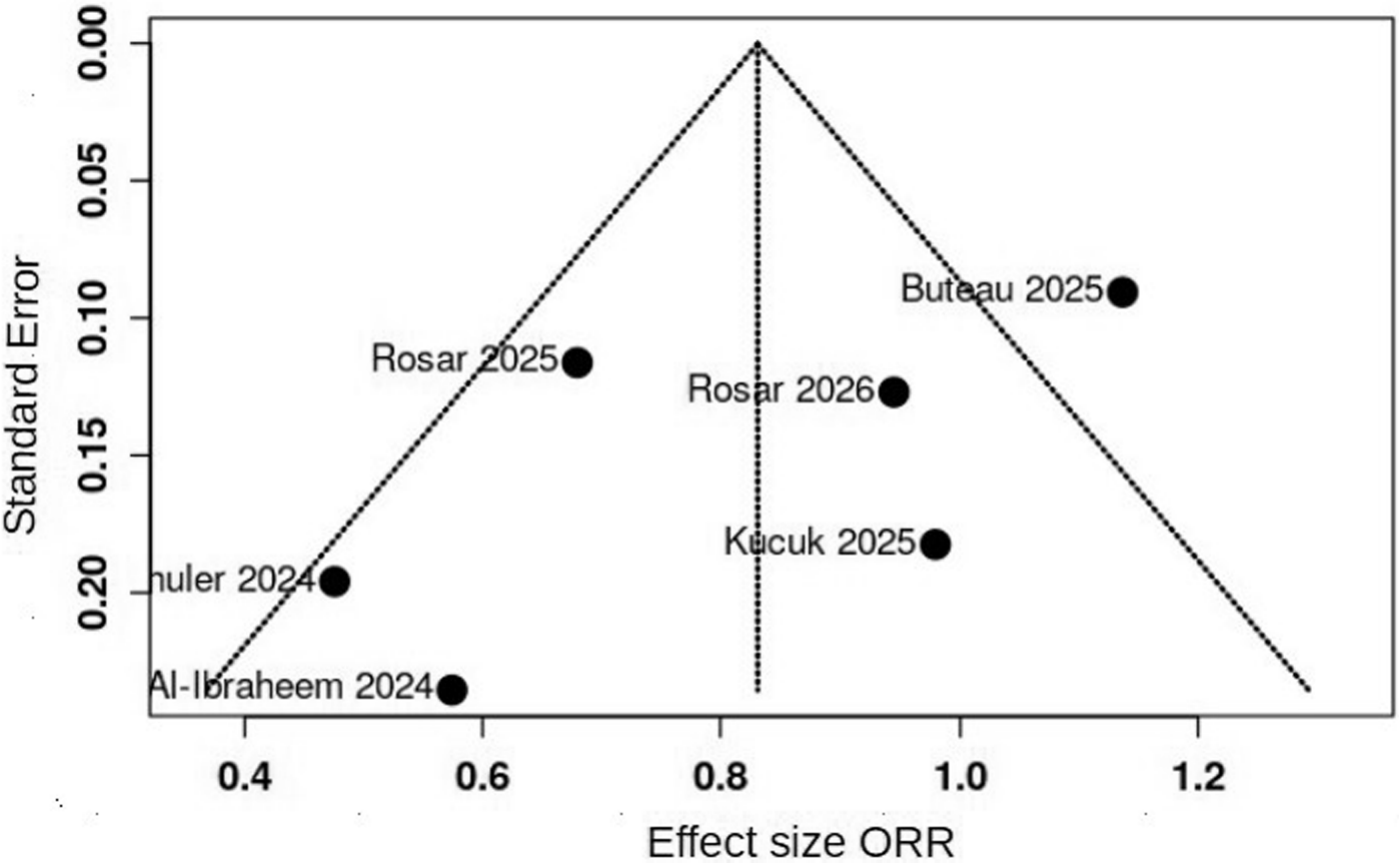
Funnel plot for the evaluation for bias assessment of studies concerning objective control rate for the assessment of biochemical efficacy of [^161^Tb]Tb-PSMA RLT.

Instead, the funnel plot for ORR does not indicate a potential publication bias (Figure 5). The Egger’s test does not support the presence of funnel plot asymmetry (intercept: −3.57, 95% CI: −7.51–0.36, *t*: −1.78, *p*-value: 0.15).

**Figure 6:**
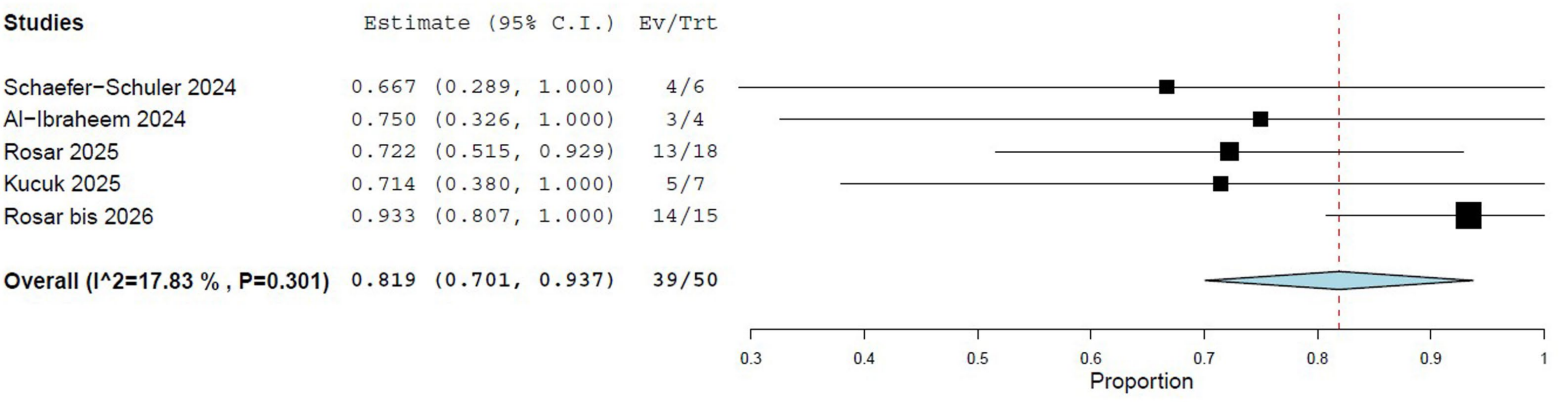
Forest plot of disease control rate for the assessment of biochemical efficacy of [^161^Tb]Tb-PSMA RLT including only studies receiving prior PSMA-RLT.

**Figure 7:**
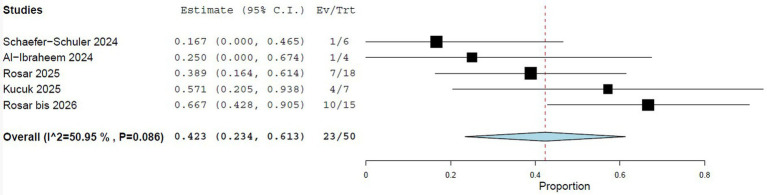
Forest plot of objective response rate for the assessment of biochemical efficacy of [^161^Tb]Tb-PSMA RLT including only studies receiving prior PSMA-RLT.

A subanalysis excluding one study focus on PSMA-RLT-naïve (treated after ARPI/taxane failure rather than after prior ^177^Lu/225Ac-PSMA failure) (12), the pooled DCR was 81.9% (95% CI: 70–94%) without significant heterogeneity (I^2^ = 17.83%, *p* = 0.301) (Figure 6) and the pooled ORR was 50.95% (95% CI: 23–61%) with a moderate heterogeneity (I^2^ = 50.95%, *p* < 0.001) (Figure 7).

Three studies originated from the same center (10, 13, 15), so a partial patient overlap cannot be completely excluded. However, these reports evaluated different clinical cohorts or treatment stages whenever explicitly stated by the original authors.

## Discussion

This systematic review and meta-analysis provides the first quantitative synthesis of the currently available evidence regarding the efficacy and safety of [^161^Tb]Tb-PSMA RLT in patients with mCRPC. By pooling data from six studies including 80 patients and 291 treatment cycles, our analysis demonstrated encouraging antitumor activity together with a manageable safety profile. In particular, the pooled DCR was 83.9%, while the pooled ORR reached 50.2%, suggesting that [^161^Tb]Tb-PSMA RLT may represent a promising therapeutic option for patients with advanced disease, including those who have exhausted standard treatment alternatives. Considering the aggressiveness of this condition and the small therapeutic options, this RLT could be a valid treatment (16).

The rationale for the clinical development of ^161^Tb is based on its favorable physical characteristics (5, 6). Similar to ^177^Lu, ^161^Tb emits *β*-particles suitable for therapeutic purposes; however, it also releases a substantial number of low-energy conversion and Auger electrons (17). These additional emissions result in a higher localized energy deposition at the cellular and subcellular level, potentially improving the irradiation of isolated tumor cells and microscopic disease deposits while preserving surrounding healthy tissues. Experimental and dosimetric studies have consistently suggested a therapeutic advantage of ^161^Tb over ^177^Lu, particularly in small-volume lesions and micrometastatic disease (18). Our findings provide preliminary clinical support for these theoretical advantages and indicate that the transition from preclinical promise to clinical efficacy may be feasible.

An important point of the present analysis is that most included patients had advanced pretreated disease. In almost all studies, [^161^Tb]Tb-PSMA RLT was administered after progression following previous PSMA-targeted radionuclide therapies, including [^177^Lu]Lu-PSMA and, in some cases, [^225^Ac]Ac-PSMA. Therefore, the observed response rates are particularly noteworthy because they were achieved in a population characterized by limited therapeutic options and potentially resistant tumor biology. The ability of ^161^Tb-based treatment to induce disease control in this setting suggests that the different radiation spectrum of ^161^Tb may partially overcome mechanisms limiting the efficacy of conventional *β*-emitting radionuclides. Nevertheless, direct evidence supporting superiority over ^177^Lu remains limited, and prospective comparative studies are needed to clarify the magnitude of any additional clinical benefit.

The safety profile observed in our review was generally favorable. Although adverse events occurred in approximately 41% of patients, most toxicities were low grade and manageable. Hematological toxicity, particularly anemia, represented the most common adverse event, while fatigue was the most frequently reported clinical side effect. These findings are largely consistent with the known toxicity profile of PSMA-targeted radionuclide therapies and do not suggest the emergence of unexpected safety concerns associated with ^161^Tb. Importantly, despite the additional emission of conversion and Auger electrons, no substantial increase in severe toxicity was observed. This observation supports the hypothesis that the enhanced radiobiological properties of ^161^Tb may be achieved without a significant compromise in tolerability (2, 3). Nevertheless, longer follow-up periods and larger patient cohorts are required to better characterize potential cumulative toxicities, especially in patients receiving multiple treatment cycles.

Our results should also be interpreted in the context of the broader landscape of PSMA-targeted radionuclide therapies. Recent meta-analyses have demonstrated the efficacy of [^177^Lu]Lu-PSMA RLT in mCRPC, with PSA responses and disease control translating into improved survival outcomes (19–21). Furthermore, several emerging radionuclides, both in the diagnostic field (22) and in therapeutic scenario, including *α*-emitters and alternative *β*-emitters, are currently under investigation (6). In this evolving scenario, ^161^Tb may occupy a unique position because it combines the established clinical experience of β-emitting therapies with additional Auger electron-mediated cytotoxicity. Consequently, it may offer a favorable balance between efficacy and safety, although definitive comparisons with established agents remain unavailable. Compared with [^177^Lu]Lu-PSMA, [^161^Tb]Tb-PSMA possesses nearly identical chemical properties but emits a substantially greater number of low-energy conversion and Auger electrons. These emissions increase the absorbed dose at the cellular level and may improve the treatment of microscopic disease while maintaining a similar dosimetric profile for larger lesions. Current clinical evidence suggests that the safety profile of [^^161^Tb]Tb-PSMA is comparable to that reported for [^177^Lu]Lu-PSMA, whereas its efficacy appears promising even in heavily pretreated patients, including those previously exposed to Lu-177 therapy. Nevertheless, direct head-to-head comparative studies are currently lacking, and it remains uncertain whether the theoretical radiobiological advantages of terbium-161 will translate into superior progression-free survival, overall survival, or quality of life. The greatest clinical benefit may ultimately be observed in patients with low-volume metastatic disease or micrometastatic tumor burden, where Auger electron emission is expected to provide the greatest therapeutic advantage.

An important aspect not addressed by the currently available studies is the impact of [^161^Tb]Tb-PSMA RLT on patient-reported outcomes and quality of life (QoL). In patients with advanced mCRPC, preservation or improvement of QoL represents a major therapeutic goal, particularly considering the palliative intent of many treatments. Marandino et al. (23) highlighted the inconsistent assessment and reporting of QoL endpoints in phase III prostate cancer trials, emphasizing the need for standardized integration of patient-reported outcomes into clinical research. Similar considerations apply to studies investigating novel radioligand therapies. Future prospective trials evaluating [^161^Tb]Tb-PSMA should therefore include validated QoL questionnaires to provide a more comprehensive assessment of clinical benefit beyond tumor response and toxicity.

Several limitations of this study should be acknowledged. First, the number of available studies and included patients remains limited, reflecting the early stage of clinical development of [^161^Tb]Tb-PSMA RLT. Second, considerable heterogeneity existed among the included studies regarding patient selection, prior treatments, administered activities, number of treatment cycles, radiopharmaceutical compounds, and response assessment methods. This variability likely contributed to the significant heterogeneity observed in the pooled ORR analysis. Third, most studies were single-center investigations with relatively short follow-up periods and without control groups. Fourth, survival outcomes such as progression-free survival and overall survival could not be pooled because of insufficient and heterogeneous reporting. Finally, publication bias cannot be completely excluded, given the limited number of available studies and the predominance of early exploratory clinical experiences.

The inability to perform pooled analyses of progression-free survival and overall survival limits the clinical interpretation of our findings. While disease control and objective response represent useful early indicators of therapeutic activity, they cannot be considered surrogates for long-term clinical benefit. Consequently, the true impact of Tb-PSMA RLT on survival remains uncertain and should be clarified in future prospective studies with standardized survival reporting.

## Conclusion

Despite these limitations, the present work provides a comprehensive overview of the currently available evidence and highlights the potential clinical value of [^161^Tb]Tb-PSMA RLT. The encouraging efficacy signals observed in pretreated mCRPC patients, together with an acceptable toxicity profile, support further clinical development of this therapeutic approach. Future multicenter prospective trials, ideally including direct comparisons with [^177^Lu]Lu-PSMA and other emerging radionuclide therapies, are warranted to better define the optimal clinical positioning of ^161^Tb-based treatment, identify patients most likely to benefit, and establish its impact on long-term clinical outcomes.

## Data Availability

The data analyzed in this study will be made available by the authors upon request. Requests to access these datasets should be directed to domenico.albano@unibs.it.
