# Supplementary material for: Efficacy and safety of [161Tb]Tb-PSMA radioligand therapy in metastatic castration resistant prostate cancer: a systematic review and meta-analysis
Source: Front Med (Lausanne). 2026 Aug 5;13:1925657. doi: 10.3389/fmed.2026.1925657 (PMC13485755; doi:10.3389/fmed.2026.1925657)
Supplement: Supplementary file 2 [file Table_2.DOCX]

**Supplemental Table 2** Results of Methodological Quality evaluation by NIH evaluation tool

| **Item** | **1** | **2** | **3** | **4** | **5** | **6** | **7** | **8** | **9** | **10** | **11** | **12** | **13** | **14** | **Numerical score** | **Quality score** |
| --- | --- | --- | --- | --- | --- | --- | --- | --- | --- | --- | --- | --- | --- | --- | --- | --- |
| Schaefer-Schuler et al. [10] | yes | yes | x | yes | no | yes | yes | yes | yes | yes | yes | no | yes | no | 10 | good |
| Al-Ibraheem et al. [11] | yes | yes | x | yes | no | yes | yes | yes | yes | yes | yes | no | yes | no | 10 | good |
| Buteau et al. [12] | yes | yes | x | yes | no | yes | yes | yes | yes | yes | yes | no | yes | no | 10 | good |
| Rosar et al. [13] | yes | yes | x | yes | no | yes | yes | yes | yes | yes | yes | no | yes | yes | 11 | good |
| Kucuk et al. [14] | yes | yes | x | yes | yes | yes | yes | yes | yes | yes | yes | no | yes | yes | 12 | good |
| Rosar et al. [15] | yes | yes | x | yes | no | yes | yes | yes | yes | yes | yes | no | yes | no | 10 | good |
